# Exploring the Causes of Wastage of Blood and Its Components in a Tertiary Care Hospital Blood Bank

**DOI:** 10.7759/cureus.20500

**Published:** 2021-12-18

**Authors:** Farrah Bashir, Attika Khalid, Shahbaz Iqbal, Tariq Ghafoor, Moiz Ahmed

**Affiliations:** 1 Pediatric Oncology, Combined Military Hospital, Rawalpindi, PAK; 2 Pathology, Foundation University Medical College, Rawalpindi, PAK; 3 Pathology, Fauji Foundation Hospital Rawalpindi, Rawalpindi, PAK; 4 Pathology, Excel Healthcare Laboratories (Pvt) Ltd, Karachi, PAK; 5 Stem Cell Transplant, Armed Forces Bone Marrow Transplant Centre / National Institute of Blood and Marrow Transplant, Rawalpindi, PAK; 6 Medicine, Jinnah Postgraduate Medical Centre, Karachi, PAK

**Keywords:** discarded blood, fresh frozen plasma, human immunodeficiency virus, hepatitis b, hepatitis c, platelets, red cell component, wastage

## Abstract

Background

Blood donated by healthy people is extremely important as it is integral in emergent situations. The authors aimed to address and highlight the main causes of the wastage of donated blood and its components.

Methodology

A cross-sectional study was conducted at a blood bank of a tertiary care center between January 2019 and March 2020. All the information regarding blood donated and blood components during the study period was documented on a predefined proforma. The blood bags which were seropositive, reached their shelf-life expiry, expired due to non-utilization, or quantity was non-sufficient were discarded. Blood showing any changes of either hemolysis or turbidity was also discarded. Other reasons for discarding blood units included leakage (damage to or fault in the blood bag), hemolytic reasons, or miscellaneous reasons.

Results

A total of 9308 blood donations were received as donations during the study period. Out of the total donations, 7,988 (85.8%) were subjected for component formation including red cell component (RCC), fresh frozen plasma (FFP), and platelets. A total of 23,964 components were prepared using the donated blood. Out of these 2128 (8.87%) units were discarded. Upon stratifying the discarded blood according to the type of component, it was found that platelets made up 1148 (53.9%) units, red cell component composed 324 (15.2%) units, and fresh frozen plasma composed 313 (14.7%) units of discarded blood. Seropositive was reported to be 32.3%. Of this, the red cell component made up 276 (85.2%) units.

Conclusion

The present study reported a discard rate of 8.87%. Of these, the majority was composed of platelets due to the shortest shelf life. Leakage of blood bags remained a predominant cause for the discard of blood components. Seropositivity for hepatitis B, C, and human immunodeficiency virus (HIV) was reported in almost 30% units of donated blood. Further large-scale studies should be conducted to reassess how wastage of donated blood can be minimized.

## Introduction

Transfusion of blood and blood components (RBCs, platelets, plasma, and cryoprecipitate) is an essential therapeutic intervention and the most common procedure performed in medicine at present [1]. It has become an integral part of a patient’s management in today’s health care system due to many factors like increasing life expectancy and increasing health incidents and medical awareness [2-4]. Many medical institutes in Pakistan bear loads of many transfusion-dependent conditions such as hemoglobinopathies, coagulopathies amongst which thalassemia tops the list just like its neighbor, India [1,4].

This vital health care resource has no complete substitute to date [5]. Therefore, each unit of blood and its components is precious and must be utilized judiciously with minimal wasting [6]. The demand for blood is always outpacing the supply or the stock in blood banks [1]. To manage the increasing requirements and availability of blood and its components, strict criteria should be set and followed for the proper utilization of this essential yet limited resource [5,7,8]. The success in combating this load depends upon the efficient and intellectual use of financial and material resources, keeping in view the existing conditions in respective regions [9]. Over time, blood transfusion authorities have made significant advancements in several subdivisions of blood banking to minimize wastage and ensure proper usage of blood products. Areas such as donor management, storage of blood, cross-matching, rational use of blood, and its distribution have seen many advancements and improvements [2]. New evidence-based transfusion guidelines or triggers are promoted to rationalize blood utilization and reduce harmful transfusion complications recently as well [7,10,11].

Blood components may be inadvertently wasted within the laboratory at any stage of the preparation [12]. This wastage of blood components is an important issue at all hospitals and medical centers as well [13]. Various factors can lead to wastage of a blood product, which includes shelf-life expiry, quality control issues, or seropositive [2,5,6].

Due to the scarcity of local and international data on the subject, the authors undertook the present study to explore the causes of blood component wastage in the blood bank laboratory. This present study was conducted and focused to find out the frequency as well as the causes for wastage of whole blood or components. The findings put forth can assist in guiding towards the path to make quality assurance checks and policies that can minimize blood wastage.

## Materials and methods

A cross-sectional study was conducted at a tertiary care center between January 2019 and March 2020. Ethical approval was obtained from the Institutional Review Board (IRB) of Combined Military Hospital (Reference # IRB/3290/CMH). A non-probability convenience sampling technique was applied to recruit the participants.

All the information regarding blood donated and blood components during the study period was documented on a predefined proforma. All individuals who consented to donate their blood were included in the study. The donors either volunteered or donated blood to replace blood for their own patients. Replacement donors were either relatives or friends of the patients. All donated blood during the study period was screened for any blood transmissible diseases including but not limited to hepatitis B, hepatitis C, or human immunodeficiency virus (HIV).

The blood bags which were seropositive, reached their shelf-life expiry, expired due to non-utilization, or quantity was non-sufficient were discarded. Blood showing any changes of either hemolysis or turbidity was also discarded. Other reasons for discarding blood units included leakage (damage to or fault in the blood bag), hemolytic reasons, or miscellaneous reasons. The senior medical laboratory technician made the call to discard a unit of blood. The reason for discarding the blood was documented.

The donors’ age, gender, and reason to donate were recorded. Furthermore, total blood units, blood components, discard rate, causes of discard, and seropositivity, etc. were also recorded. All data were analyzed using Statistical Package for Social Sciences (SPSS) version 26 (IBM Corporation, Armonk, NY). All categorical data were represented as frequency and percentages.

## Results

A total of 9308 blood donations were collected during the study period. Of these maximum donations were made in exchange for blood for the donors’ patients. Voluntary donors comprised only 0.23% of the total donations made during the study. Males were the predominant population to donate blood with only 145 (1.5%) female donors.

Out of the total donations, 7,988 (85.8%) were subjected for component formation including red cell component (RCC), fresh frozen plasma (FFP), and platelets. A total of 23,964 components were prepared using the donated blood. Out of these 2128 (8.87%) units were discarded. Upon stratifying the discarded blood according to the type of component, it was found that platelets made up 1148 (53.9%) units, red cell component composed of 324 (15.2%) units, and fresh frozen plasma composed 313 (14.7%) units of discarded blood (Table 1).

**Table 1:** Distribution of Discarded Blood Components According to the Causes

Components	Causes of Discarding Blood Units									Total Components
	Seroreactivity (%)	Leakage (%)	Lipaemic (%)	Shelf-life expiry (%)	Dispensed but not used (%)	Machine error (%)	RBC Contamination (%)	Quantity Not Sufficient (QNS) n (%)		
Platelets	212 (14.1%)	66 (4.4%)	29 (1.9%)	932 (62.2%)	43 (2.9%)	94 (6.3%)	54 (3.6%)	69 (4.6%)		1499 (70.44%)
Fresh Frozen Plasma	199 (65.2%)	51 (16.7%)	21 (6.9%)	-	9 (3.0%)	-	1 (0.3%)	24 (7.9%)		305 (14.33%)
Red Cell Component	276 (85.2%)	-	-	13 (4.0%)	30 (9.3%)	-	-	5 (1.5%)		324 (15.23%)
Total	687 (32.3%)	117 (5.5%)	50 (2.3%)	945 (44.4%)	82 (3.9%)	94 (4.4%)	55 (2.6%)	98 (4.6%)		2128

**Figure 1:**
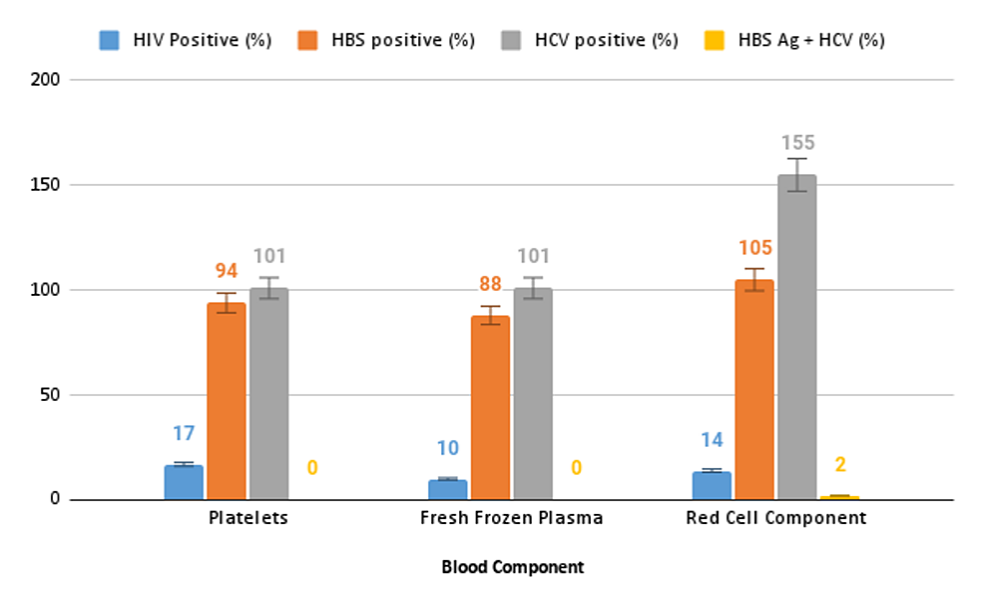
The Distribution of Discarded Blood Units According to the Seroreactivity

Seropositivity was reported to be 32.3%. Of this, the red cell component made up 276 (85.2%) units. The distribution of seroreactivity according to the different blood components is illustrated in Figure 1. The highest rates of hepatitis C virus and hepatitis B infection positivity were found in the red cell component with a frequency of 155 and 105 units, respectively. HIV infection positivity was slightly higher in platelets (Figure 1).

## Discussion

Wastage of blood and blood products is an alarming issue. The dearth of blood banking services and stringent quality requirements of blood banks has made the issue of the wastage of precious components of blood an area of concern [11,12]. This study was carried out to get an insight into the causes of discard of various blood components. This can help in assessing preventable causes and make future plans and proposals to minimize wastage.

The overall discard rate in our study was 8.87%. In many studies conducted globally a fairly similar pattern of components discard has been observed [2,14-16]. Patil et al. had a discard rate of 22.45% which is much higher than our study [14]. The reason is the underutilization of the platelets in their hospital.

The shelf life is only five days for platelets, up to 35 days for red cell components, and 36 months for fresh frozen plasma. The current study showed that of the total units discarded due to shelf-life expiry, platelets made up for more than half of them since platelets have the shortest half-life. This is in line with other literature as well [14,15]. Only 4% of red cell components were discarded due to expiry since the shelf life is longer. Shelf-life expiry for fresh frozen plasma was not reported in our study. This is seen as a global issue. Our hospital has separate pediatric oncology and adult oncology units. A ready source of platelet is a dire need for unforeseen emergencies in critical situations. For a judicious supply of this component in such conditions, a fraction of platelet expiry is both inevitable and unavoidable.

Proper donor screening and strict adherence to the donor selection guidelines would decrease the collection of such units from the donors, thereby avoiding discard of such units. In our study, we found seropositivity for hepatitis B, C, and HIV to be in 687 (32.3%) units of donated blood.

In our study leakage of blood bags remained a predominantly occurring cause for discard of FFPs and platelets. This is amongst the most frequently occurring factors in other studies as well [3,14,17], albeit higher than our study. In a study, overall 26% of blood components were discarded due to leakage [3].

Blood bags could be leaked and ruptured during transportation or processing owing to mishandling of blood bags during collection or manufacturing errors [18-20]. It is noted that the quality of the blood bags is an extremely important factor that contributes to preventing leakage [12]. It is also possible that during centrifugation, the blood bag is damaged due to the sharp bottom junction thereby ripping the bag apart [18]. Visual inspection must be done in order to assess the integrity of the bag [20]. Furthermore, it is advisable to store fresh frozen plasma (FFP) in a cover made up of either cardboard or polystyrene to protect the contents during freezing, thawing, or handling [1]. Similarly, in order to reduce the chances of damage, the bag should be placed in a plastic bag before immersing it in lukewarm water [12].

The current study had some limitations, for instance, we only observed discard rate and its causes within the blood bank. The study did not account for the blood that was released from the bank and was never administered to the patients because either the patient expired before the blood could be administered. Another possibility is that the blood was wasted after being released from the blood bank because of malfunctioning cannulae systems or no refrigeration systems within the wards to store the blood temporarily. Further research into the matter is warranted.

Up-gradation of storage and other facilities at the bank to deal with more inventories, use of a software-based system for monitoring stock and predicting utilization, and the aforementioned information to screen donors for collection of the optimum amount of blood of required blood groups can be done to ensure optimized utilization of blood and its components. Furthermore, continual education programs to improve the performance of related staff can help prevent wastage of blood.

## Conclusions

The present study reported a discard rate of 8.87%. Of these, the majority was composed of platelets due to the shortest shelf life. Leakage of blood bags remained a predominant cause for the discard of blood components. We hope that the current study would serve as a catalyst to build the foundation of future studies that explore various ways in which blood wastage can be minimized. Several easily applicable interventions are recommended such as educational outreach, social media, and improved handling that can be effective to reduce blood wastage.
